# Europe-wide comparison regarding the first medical examination of the newborn after birth: Absence of uniform standards

**DOI:** 10.18332/ejm/188116

**Published:** 2024-06-27

**Authors:** Linda Plail, Sven Wellmann, Christian Apfelbacher, Michael Kabesch

**Affiliations:** 1 Science and Innovation Campus Regensburg (WECARE), Hospital St. Hedwig of the Order of St. John, Regensburg, Germany; 2 Department of Neonatology, University Children's Hospital Regensburg (KUNO), Hospital St. Hedwig of the Order of St. John, University of Regensburg, Regensburg, Germany; 3 Institute of Social Medicine and Health Systems Research, Faculty of Medicine, Otto von Guericke University Magdeburg, Magdeburg, Germany; 4 Department of Paediatric Pneumology and Allergy, University Children's Hospital Regensburg (KUNO), Hospital St. Hedwig of the Order of St. John, University of Regensburg, Regensburg, Germany

**Keywords:** neonatal examination, legal requirement, medical guideline, midwife, baby

## Abstract

**INTRODUCTION:**

The first medical examination of the newborn after birth plays an essential role in identifying congenital malformations and life-threatening conditions. Currently, no Europe-wide guidelines or standards for performing the first neonatal examination exist. It is unclear which professional group carries out this examination in different European countries. Additionally, there are no requirements for an examination accepted throughout Europe. The objective of this cross-sectional study was to identify the status quo of medical guidelines and legal requirements in place as well as to determine which profession carries out the first neonatal examination in European countries.

**METHODS:**

By means of a structured questionnaire, one expert survey at two international medical specialist conferences in Europe in 2019 were carried out. Participants were asked whether medical guidelines or legal requirements exist in their home country and which medical profession is recommended to perform the neonatal examination. Survey participants were delegates of national neonatal or perinatal societies. To verify statements, further neonatal experts at European level were contacted.

**RESULTS:**

A total of 51 participants from 35 countries in Europe were interviewed. Overall, 28 of 35 participating countries (80%) have published medical guidelines and 24 (69%) have legal requirements in place for the first neonatal examination. A wide range of professional groups (midwives, neonatologists, pediatricians, obstetricians, general practitioners, nurse practitioners and advanced neonatal nurse practitioners) performs the first neonatal exam. In 27 (77%) countries, midwives are the main group of examiners.

**CONCLUSIONS:**

Currently a European patchwork of different medical guidelines and legal requirements in regard to the first medical examination of the newborn after birth exists. In addition, a variety of professional groups perform the first neonatal examination. There is great potential for standardization and an expert committee could establish common European guidelines in order to ensure the best possible neonatal care throughout Europe.

## INTRODUCTION

The focus of the first neonatal examination is usually on the detection of life-threatening problems related to the transition from intra-uterine to extra-uterine life and the identification of inherited conditions and congenital malformations, which may require timely action to prevent deterioration and should be detected with a high level of sensitivity and specificity early after birth^1^. Worldwide, the medical and legal frameworks vary largely in which early prevention and screening programs are organized, due to country specific histories, different needs and different paces in transforming traditional medical approaches to modern data-driven concepts^2^.

Thus, the first physical examination of the newborn after birth is performed in each country according to national, regional, and institution-specific standards. For example, in the United Kingdom there is the newborn and infant physical examination (NIPE) screening-program handbook^3^ of the United Kingdom National Health Service. In Germany there is the federal joint committee guideline for screening exams in newborns and children^4^. In order to ensure that every newborn baby in Europe will receive the same high-quality care standards, the European Foundation for the Care of Newborn Infants (EFCNI) has initiated a transdisciplinary collaboration project to provide agreed standards for high-quality perinatal and neonatal care, whose implementation will ensure more equitable care across Europe^5^.

In some countries, neonates are discharged from birth-hospitals before a formal neonatal examination is performed^6^. In other countries, midwives rather than physicians perform neonatal examinations^7^.

Currently, no systematic evaluation of European medical standards and guidelines for the first newborn examination exists. Therefore, the aim of this study was to describe the status quo regarding medical guidelines and legal requirements in place, as well as to determine which professions carry out the first neonatal examination in the European countries.

## METHODS

### Study population

**Figure 1:**
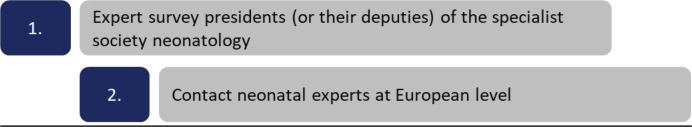
Two-step methodology of the international survey for the first medical examination of the newborn after birth

This cross-sectional study consisted of a survey with three questions and was performed in two steps (Figure 1). First, the survey was performed among delegates of national neonatal or perinatal societies attending medical specialist conferences in 2019, the meeting of the European Board of Neonatology at the 3rd jENS congress in Maastricht^8^. (n=40 participants) and at the 27th European Workshop of Neonatology 2019 in Rotterdam^9^.(n=32 participants). The delegates of the respective two conferences were presidents, vice-presidents, or representatives of national neonatal or perinatal societies from almost all European countries with a professional background in pediatrics. To facilitate the survey, delegates had the opportunity to clarify uncertainties about the survey immediately. It was explained to the participants that providing contact details was voluntary and that the results would be evaluated anonymously. In order to get responses from as many countries in Europe as possible, additional experts in neonatology were contacted from those countries not reached at the two conferences. This was done via the office of the European Society for Pediatric Research (ESPR). Second, to double check statements for validity and comprehensiveness, further neonatology experts were interviewed when answers were missing or ambiguous, and from countries not represented at both conferences.

### Measures and assessment

The survey consisted of three questions addressing the first medical examination of the newborn after birth:

1. Do medical guidelines in your country exist?
2. Do legal requirements in your country exist?
3. According to your medical guideline or legal requirements, which medical profession is allowed/recommended to perform the neonatal examination?

The questions were validated beforehand and understandability was tested in a pretest with neonatologist from three different European countries. Participants received information on the definition of medical guidelines and legal requirements at the two conferences as follows: Medical guidelines are systematically developed recommendations to support the decision-making of physicians, other healthcare professionals, and patients. They reflect the current state of best medical knowledge and strive to improve medical care without being directly legally binding. In contrast, legal requirements are legally binding requirements based on legal principles; they regulate certain aspects of medical care.

### Statistical analysis

The data collected on paper were recorded using the software Qnome (Maganamed GmbH, Regensburg, Germany). For all question categories, no statistically significant difference between answers from the two time points (first and second congress) were observed by chi-squared test and Fisher’s exact test, and results of the two were therefore analyzed as one dataset. The data were analyzed descriptively. Empirical data were prepared using tables, key figures, and graphics.

## RESULTS

**Figure 2:**
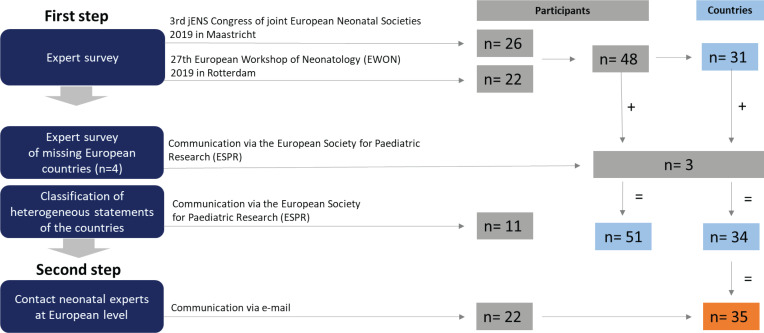
Return of the survey of the international survey for the first medical examination of the newborn after birth

**Figure 3:**
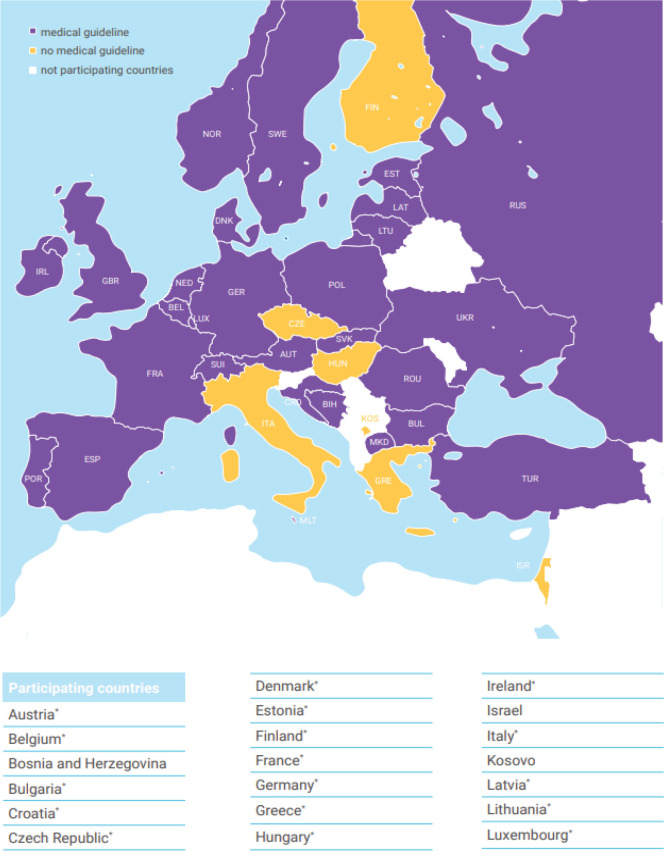
Availability of medical guidelines for the first medical examination of the newborn after birth across Europe

**Figure 4:**
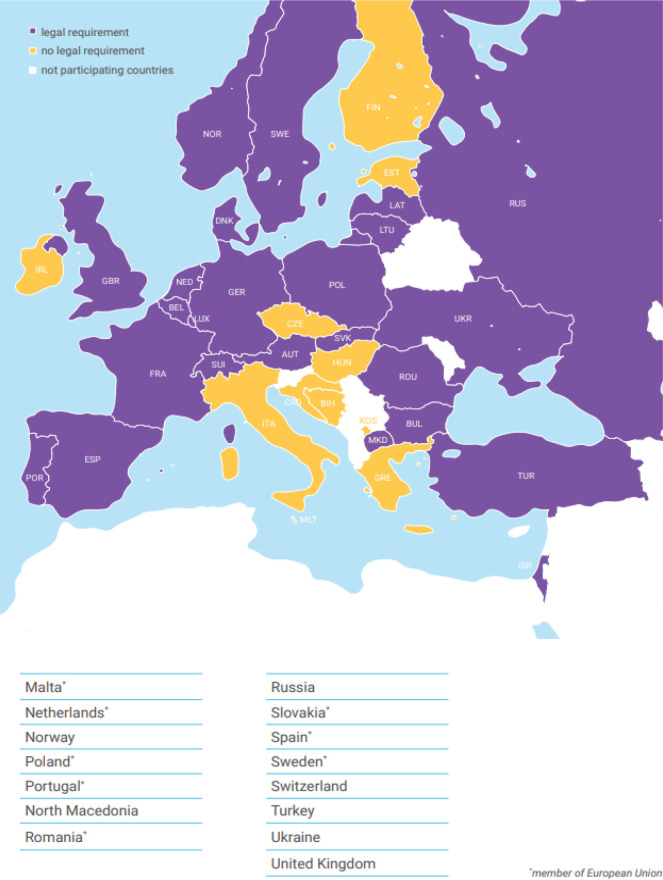
Availability of legal requirements for the first medical examination of the newborn after birth across Europe

A total of 48 representatives of the European national neonatology societies and several neonatology experts from 31 countries took part in the survey at two conferences (Figure 2). More than one answered questionnaire was obtained from Austria (2), Bulgaria (2), Denmark (2), Germany (2), Portugal (2), Ukraine (2), United Kingdom (2), Holland, Turkey, Bulgaria (3) and Greece (4). Delegates from 11 countries made heterogeneous statements and filled in questionnaires incorrectly or incomplete. These study participants were contacted via the office of the European Society for Pediatric Research (ESPR) to resolve and clarify issues. Neonatology experts from European countries not represented at the two conferences were approached via the ESPR and asked to fill in questionnaires. Thus, information from Hungary, Norway and Italy was obtained. In total, questionnaires from 35 different countries were received, including 25 out of 27 member states of the European Union (Figures 3 and 4). Subsequently, all represented delegates were contacted and asked to provide national medical guidelines and/or legal requirements from their respective countries in writing, and from 13 countries guidelines or legal requirements could be collected and documented.

According to study participants, 28 of 35 European countries (80%) have medical guidelines for the first neonatal examination in place. For the following countries no such medical guidelines exist, Czech Republic, Finland, Greece, Hungary, Israel, Italy, and Kosovo (Figure 3). Legal requirements exist in 69% of the assessed countries according to study participants, while no legal requirements for the first neonatal exam exist in Bosnia and Herzegovina, Croatia, Czech Republic, Estonia, Finland, Greece, Hungary, Ireland, Italy, Kosovo, and Malta (Figure 4).

**Figure 5:**
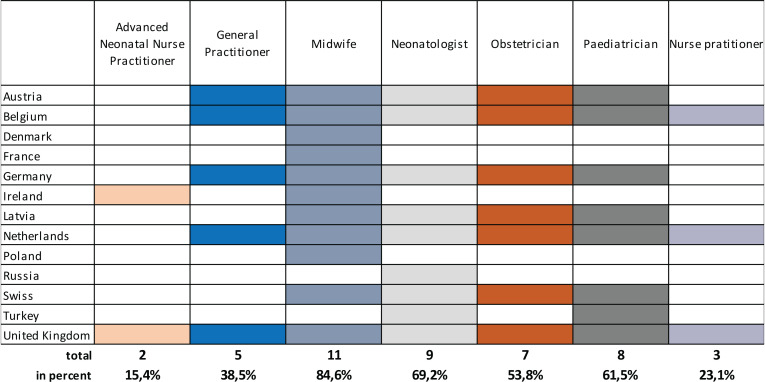
First neonatal exam immediately after birth: Recommended professional group according to national standards across Europe

In total, 13 participants in the survey provided information about the published guidelines for the first examination of the newborn. As a result, the experts’ statements could only be verified in these 13 countries. We evaluated which medical profession carries out newborn examinations in the different countries. This analysis was restricted to countries for which country-specific guidelines or legal requirements were received. In four countries, only one medical profession performs the first newborn examination, whereas in Denmark, France, and Poland only midwives perform the examination; in Russia only pediatricians perform the first medical examination of the newborn. In all other countries, at least two different professional groups are involved. However, in 85% of countries, the occupation group of midwives is the most frequently involved occupation group at the first neonatal exam, followed by neonatologists with 70% and pediatricians with 62% (Figure 5).

## DISCUSSION

Our survey among representatives of national neonatal societies and other experts in neonatology showed that a majority of, but not all European countries have published medical guidelines or legal requirements for the first medical examination of the newborn after birth in place and that predominately midwives are performing the first examination.

In 2016, the WHO published standards for improving quality of maternal and newborn care in health facilities^10^. These standards provide priorities for reducing maternal and perinatal mortality, define basic routine care standards immediately after birth, and define input and output quality measures with the aim to avoid preventable complications. These quality measures are basic, including temperature management, sterile cord clamping, skin-to-skin contact, initiation of breastfeeding, and do not specify medical examination of the neonate^10^. However, a common medical guideline for the first neonatal examination of newborns could promote the harmonization of medical practice by establishing common standards and criteria for the examination and diagnosis of conditions^11^. Furthermore, a common guideline may facilitate collaboration and exchange of knowledge and experiences among health professionals by creating a common understanding of evidence-based medicine.

It is well known that socioeconomic factors such as parents’ income, education and occupation have a significant influence on children’s health. It is important to understand the causes and manifestations of inequalities and needs of vulnerable groups in order to develop evidence-based policies and practices to address social problems^12^. In the future, it will continue to be important to address health inequalities at birth, if not earlier, to provide equal opportunities for the next generation from the start, as poor quality of care inevitably leads to higher morbidity and mortality^11^.

A consensus on how to perform neonatal screening examinations could be a first step in this direction. However, differences in terms and definitions used in the European healthcare systems and in the care of newborns, may provide challenges in developing common standards^5^. Different time points and procedures are summarized under the term ‘first neonatal examination’ or ‘baby check’ across countries. Since most medical guidelines and regulations exist in the respective national language, the first step is to collect and translate these documents into a common language to enable international experts to compare procedures between countries. Here we received documents from 13 countries and realized that this process (collect and translate) is quite cumbersome. To the best of our knowledge, there is no platform or initiative existing that collects, evaluates, or compares medical guidelines for the first neonatal examination of newborns in Europe.

### Limitations

We cannot exclude the possibility that guidelines exist in more countries than reported here. In fact, guidelines may exist but they may have been unknown to the respondents of the survey we conducted. This could indicate that healthcare professionals are either not aware of guidelines, find themselves overwhelmed, or do not want to follow guidelines. It would therefore be desirable if there was a European register for national guidelines. Another limitation is that it was not possible to obtain the relevant guidelines in writing to accompany the respondents’ statements. Therefore, the respondents’ answers to the question: ‘Which medical profession is allowed/recommended to perform the neonatal examination’, could be verified in just 13 out of 28 countries.

## CONCLUSIONS

The creation of international medical guidelines can be challenging due to the heterogeneity of health systems, different cultural and language barriers, different medical practices, interests, evidence base and legal framework in Europe. To establish a common standard, it is essential to set up coordinating organizations and networks at the European level so that language barriers are eliminated and development is promoted.

Currently a European patchwork of different medical guidelines and legal requirements exists. In addition, a range of professional groups performs the first neonatal examination. There is great potential for standardization and an expert committee could establish common European guidelines to ensure the best possible neonatal care throughout Europe.

## Data Availability

The data supporting this research are available from the authors on reasonable request.
